# Inhibitor of Sarco/Endoplasmic Reticulum Calcium-ATPase Impairs Multiple Steps of Paramyxovirus Replication

**DOI:** 10.3389/fmicb.2019.00209

**Published:** 2019-02-13

**Authors:** Naveen Kumar, Nitin Khandelwal, Ram Kumar, Yogesh Chander, Krishan Dutt Rawat, Kundan Kumar Chaubey, Shalini Sharma, Shoor Vir Singh, Thachamvally Riyesh, Bhupendra N. Tripathi, Sanjay Barua

**Affiliations:** ^1^ National Centre for Veterinary Type Cultures, ICAR-National Research Centre on Equines, Hisar, India; ^2^ Department of Biotechnology, GLA University, Mathura, India; ^3^ Department of Veterinary Physiology and Biochemistry, Lala Lajpat Rai University of Veterinary and Animal Sciences, Hisar, India

**Keywords:** SERCA, virus replication, paramyxovirus, antiviral drug resistance, host-targeting antiviral agents

## Abstract

Sarco/endoplasmic reticulum calcium-ATPase (SERCA) is a membrane-bound cytosolic enzyme which is known to regulate the uptake of calcium into the sarco/endoplasmic reticulum. Herein, we demonstrate for the first time that SERCA can also regulate virus replication. Treatment of Vero cells with SERCA-specific inhibitor (Thapsigargin) at a concentration that is nontoxic to the cells significantly reduced Peste des petits ruminants virus (PPRV) and Newcastle disease virus (NDV) replication. Conversely, overexpression of SERCA rescued the inhibitory effect of Thapsigargin on virus replication. PPRV and NDV infection induced SERCA expression in Vero cells, which could be blocked by Thapsigargin. Besides inducing enhanced formation of cytoplasmic foci, Thapsigargin was shown to block viral entry into the target cells as well as synthesis of viral proteins. Furthermore, NDV was shown to acquire significant resistance to Thapsigargin upon long-term passage (P) in Vero cells. As compared to the P0 and P70-Control, the fusion (F) protein of P70-Thapsigargin virus exhibited a unique mutation at amino acid residue 104 (E104K), whereas no Thapsigargin-associated mutations were observed in HN gene. To the best of our knowledge, this is the first report describing the virus-supportive role of SERCA and a rare report suggesting that viruses may acquire resistance even in the presence of an inhibitor that targets a cellular factor.

## Introduction

The control strategies against pathogens have classically relied upon targeting essential proteins of the pathogens (Kumar et al., 2011b). High mutation rates in viral genome allows the virus to become resistant to antiviral drugs and preexisting immunity (Andino and Domingo, 2015). Classically, antiviral drugs have been developed by directly targeting viral proteins (Chen et al., 2016). Due to high mutation rates, viruses with mutations at the druggable targets are selected for resistance. The rise in incidence of drug resistance has prompted a shift in the development of novel antiviral drugs (Ludwig et al., 2006). Viruses are obligate intracellular parasites that are highly dependent on the host. Host responses are equally important in determining the actual outcome of a disease. Upon viral infection, numerous cellular factors are dysregulated (increased or decreased expression); some of these host factors facilitate virus replication (proviral), whereas others may have antiviral function (Griffiths et al., 2013). Proviral host factors may serve as targets for the development of novel antiviral therapeutics (Kumar et al., 2008, 2011a,b).

Protein phosphorylation and dephosphorylation, mediated respectively *via* kinases and phosphatases, are ubiquitous cellular regulatory mechanisms during signal transduction which determines key cellular processes such as growth, development, transcription, metabolism, apoptosis, immune response, and cell differentiation (Coito et al., 2004). Kinome, the protein kinase complement of the human genome, completed in 2002, identified 518 protein kinase genes. These kinases have been shown to play a key role in cancer and many other diseases (Coito et al., 2004) including viral infections (Nousiainen et al., 2013), making these proteins potential drug targets.

In vertebrates, there are three families of P-type Ca^2+^-ATPases, which regulate homeostasis of intracellular Ca^2+^ level. Plasma membrane Ca^2+^-ATPase (PMCA), sarco/endoplasmic reticulum calcium-ATPase (SERCA), and secretory pathway calcium ATPAse (SPCA) are located in the plasma membrane, endoplasmic reticulum, and Golgi apparatus, respectively (Feng and Rao, 2013). SERCA transports Ca^2+^ from cytosol to the double membrane-bound (endoplasmic reticulum) intracellular compartments (Inesi et al., 2005; Arruda et al., 2007; Clapham, 2007; Primeau et al., 2018). SERCA is also involved in other cellular functions such as signal transduction, apoptosis, exocytosis (Kudla et al., 2010), cell motility (Qi et al., 2007), and transcription (Flavell and Greenberg, 2008). There are three genes (ATP2A1–3) in vertebrates that code for three SERCA isoforms, namely SERCA1–3 (Wuytack et al., 2002; Altshuler et al., 2012). Each of these genes undergoes alternative splicing and hence results in 10 SERCA proteins (two each from SERCA1 and 2 and six from SERCA3) (Martin et al., 2002). While some of these isoforms/variants are ubiquitously expressed in most cell types (SERCA2), others show a range of cell type-specific expression patterns (de Meis et al., 2005; Arruda et al., 2007; Altshuler et al., 2012). The role of these Ca^2+^-ATPases in virus replication is only beginning to be appreciated. Whereas the role of SERCA and PMCA in virus replication remains unknown, a recent study suggests that SPCA1 supports virus replication (Hoffmann et al., 2017).

Previously, we screened a library of kinase and phosphate inhibitors for their antiviral potential and identified several hits against influenza A viruses (Kumar et al., 2011a). Herein, we also screened a library of these chemical inhibitors for their antiviral effects against paramyxovirus-morbillivirus [(peste des petits ruminants virus (PPRV)] and avulavirus [(Newcastle disease virus (NDV)]. SERCA inhibitor (Thapsigargin) was identified as one candidate that blocked NDV and PPRV replication. We show that Thapsigargin can block multiple steps of paramyxovirus replication, thus revealing SERCA as a potential target for the development of antiviral therapeutics.

## Materials and Methods

### Cells and Viruses

Vero (African green monkey kidney), 293 T (human embryonic kidney), MDBK (Madin-Darby bovine kidney), HeLa, and goat kidney cells were grown in Dulbecco’s Modified Eagle’s Medium (DMEM) supplemented with antibiotics and 10% heat-inactivated fetal bovine serum. PPRV, NDV, buffalopox virus (BPXV), and bovine herpesvirus 1 (BHV-1) were available in our laboratory and have been described elsewhere (Kumar et al., 2016). Viral titers were determined by plaque assay, as previously described (Kumar et al., 2016).

### Inhibitor

Thapsigargin (SERCA inhibitor) was procured from Sigma (Catalog Number-T9033, Steinheim, Germany). Thapsigargin is a noncompetitive inhibitor of SERCA. It is extracted from a plant *Thapsia garganica* and structurally classified as a sesquiterpene lactone (Rasmussen et al., 1978).

### Antibodies

SERCA2 ATPase Antibody (MA3-919) was procured from Invitrogen (Carlsbad, USA). HA Tag Monoclonal Antibody was procured from Thermo Fisher Scientific (Rockford, USA). Anti-PPRV serum that predominantly reacts with PPRV HN, F, and M proteins and anti-NDV serum that predominantly reacts with NDV F and HN proteins (in Western blot) are described elsewhere by our group (Khandelwal et al., 2017). Secondary fluorescein isothiocyanate (FITC)-conjugated anti-rabbit antibody and secondary tetramethylrhodamine isothiocyanate (TRITC)-conjugated anti-mouse antibody were purchased from Sigma (Steinheim, Germany).

### MTT Assay

Cytotoxicity of Thapsigargin was analyzed in MTT assay, as previously described (Khandelwal et al., 2017).

### Antiviral Efficacy

Vero/MDBK cells were infected with respective viruses at MOI = 0.1 in the presence of 0.5 μM Thapsigargin or vehicle control (0.05% DMSO). Progeny virus particles released in the supernatants were quantified by plaque assay.

### Effect of Thapsigargin on NDV Replication *in ovo*

Specific pathogen-free (SPF) embryonated chicken eggs were procured from Immunetic Life Sciences Pvt. Ltd. Una, India. LD_50_ of Thapsigargin was determined by inoculating five-fold serial dilutions of Thapsigargin (concentration ranging from 6,250–10 ng/egg) or vehicle control, in 10-day-old embryonated SPF eggs, in a total of 100 μl volumes *via* allantoic route. Eggs were examined for viability of the embryos up to 5 days post-inoculation to determine the LD_50_ by the Reed-Muench method.

To analyze the effect of Thapsigargin on NDV replication *in ovo*, SPF eggs were infected with 100 μl of NDV (HA titer = 2^7^) in 10-day-old embryonated SPF eggs *via* allantoic route. The allantoic fluid was collected at 6 and 96 h post-infection and quantified for NDV by hemagglutination assay (chicken red blood cells) as described previously (Kumar et al., 2016).

### Virucidal Activity

Virus suspensions (PPRV/NDV) containing ~10^6^ plaque forming units (pfu) were incubated in serum-free medium containing either DMSO or 10-fold dilutions of the Thapsigargin, for 1.5 h at 37°C. Thereafter, the samples were chilled at 4°C and diluted by 10^−3^-, 10^−4^-, and 10^−5^-fold before being applied onto Vero/MDBK cells in six-well plates for plaque assaying. The results were plotted as relative infectivity of virions against concentrations of the compound used.

### Overexpression of SERCA

HeLa/goat kidney cells were transfected in 24-well plates, in triplicates with either 1 μg of pCR3 (empty vector) or with pCR3-SERCA2.HA (SERCA with HA tag at 3′ end) using Lipofectamine 3000 transfection reagent as per the instructions of the manufacturer. At 48 h post-transfection, cells were infected with virus (NDV to HeLa cells and PPRV to goat kidney cells) at MOI of 1. Virus particles released in the supernatant at 24 h post-infection (hpi) (NDV) or 96 hpi (PPRV) were quantified by plaque assay.

### Attachment Assay

The attachment assay was performed as described previously (Khandelwal et al., 2014). Briefly, Vero cells were preincubated with 0.5 μM Thapsigargin or vehicle control for 1 h and then infected with PPRV or NDV at MOI of 5 for 2 h at 4°C. The cells were then washed six times with PBS, and the cell lysates were prepared by rapid freeze-thaw method. The virus titers were determined by plaque assay.

### Entry Assay

Vero cell monolayers were prechilled to 4°C and infected with the respective viruses at MOI of 5 in Thapsigargin-free medium for 1 h at 4°C to permit attachment, followed by washing and addition of fresh medium containing 0.5 μM Thapsigargin or vehicle control (0.05% DMSO). Entry was allowed to proceed at 37°C for 1 h after which the cells were washed again with PBS to remove any extracellular viruses and incubated with cell culture medium without any inhibitor. The progeny virus particles released in the cell culture supernatants in the treated and untreated cells were titrated by plaque assay.

### qRT-PCR

The levels of viral RNA in the infected cells were quantified by quantitative real-time PCR (qRT-PCR). Viral RNA/DNA Purification Kit (Thermo Scientific, Vilnius, Lithuania) was used for extraction of viral RNA from the infected cell lysate. cDNA was synthesized as per the protocol described by the manufacturer (Fermentas, Hanover, USA) using random hexamer primer. The resulting cDNA was stored at −20°C until use. qRT-PCR was carried out with a 20 μl reaction mixture containing gene-specific primers, template and Sybr green DNA dye (Promega, Madison, USA), and run on LineGene 9600 Bioer Real-Time PCR Detection Systems. Thermal cycler conditions were as follows: a denaturation step of 5 min at 94°C followed by 40 cycles of amplification (30 s at 94°C, 30 s at 55°C, and 30 s at 72°C). The levels of viral RNA, expressed as threshold cycle (Ct) values, were analyzed to determine relative fold change in RNA copy number as described previously (Kumar et al., 2016). The primers used for qRT-PCR were as follows: NDV (F gene) (forward primer: 5′-CAGCTGCAGGGATTGTGGT-3′ and reverse primer: 5′-TCTTTGAGCAGGAGGATGTTG-3′) and PPRV (nucleoprotein gene) (forward primer: 5′-ACAGGCGCAGGTTTCATTCTT-3′ and reverse primer: 5′-GCTGAGGATATCCTTGTCGTT-3′).

### Viral Protein Synthesis

Vero cells were either mock-infected or infected with PPRV or NDV at MOI of 10 for 3 h followed by washing with PBS and addition of 0.5 μM Thapsigargin or vehicle control (0.05% DMSO). The cells were scrapped at 9 and 20 hpi, respectively, for NDV and PPRV to prepare the cell lysate. Viral proteins were probed by Western blot analysis.

### Virus Release Assay

Virus release assay was performed as described previously (Kumar et al., 2011a). Briefly, confluent monolayers of Vero cells were infected with PPRV or NDV, in triplicates, for 2 h at MOI of 5 followed by washing and addition of fresh media. At 36 and 10 hpi, respectively, for PPRV and NDV, cells were washed six times with chilled PBS followed by addition of fresh medium containing 0.5 μM Thapsigargin or vehicle control. Virus released at 1 and 2 h (PPRV) or 30 min and 1 h (NDV) was quantified by plaque assay.

### Immunofluorescence Assay

Vero cells were grown in chamber slides at ~20% confluency and infected with PPRV/NDV at MOI of 5 for 2 h followed by washing with PBS and replacement with fresh medium. Thapsigargin was applied at 3 and 10 hpi, respectively, in NDV- and PPRV-infected cells. The intracellular localization of viral proteins in the virus-infected cells was detected by immunofluorescence assay. Briefly, cells were fixed with 4% paraformaldehyde for 15 min and blocked by 1% bovine serum albumin for 30 min at room temperature. After washing with PBS, cells were stained with primary antibody (rabbit anti-NDV/rabbit anti-PPRV or mouse SERCA2 ATPase) for 30 min in the presence of 0.2% saponin. Thereafter, cells were washed three times with PBS and incubated with a secondary fluorescein isothiocyanate-conjugated anti-rabbit antibody or secondary rhodamine-conjugated anti-mouse antibody in the presence of 0.2% saponin for 30 min. After being washed again with PBS, the cells were mounted with a medium containing 4,6-diamidino-2-phenylindole (DAPI; Sigma) and examined by fluorescence microscopy.

### Selection of Thapsigargin-Resistant Viral Mutants

NDV was sequentially passaged (70 passages) in Vero cells in the presence of either 0.25 μM Thapsigargin or vehicle control (0.05% DMSO). At each passage, confluent monolayers of Vero cells were infected with NDV, washed five times with PBS before a fresh aliquot of DMEM was added, and incubated for 72–96 h or until the appearance of cytopathic effect (CPE) in ≥75% cells. The virus released in the supernatant was termed as passage 1 (P1). The virus was quantified by plaque assay, and it (at MOI of 0.01) was used in the second round of infection, which was termed as passage 2 (P2). Seventy passages of virus infection were similarly carried out. In order to study the relative resistance against Thapsigargin at various passages, Thapsigargin-passaged and DMSO-passaged viruses were used to infect Vero cells at MOI = 0.1 and grown in the presence of either 0.05% DMSO or 0.5 μM Thapsigargin. The virus released in the supernatant was quantified by plaque assay, and fold-inhibition level was determined.

## Results

### SERCA Inhibitor Blocks Paramyxovirus Replication

**Figure 1:**
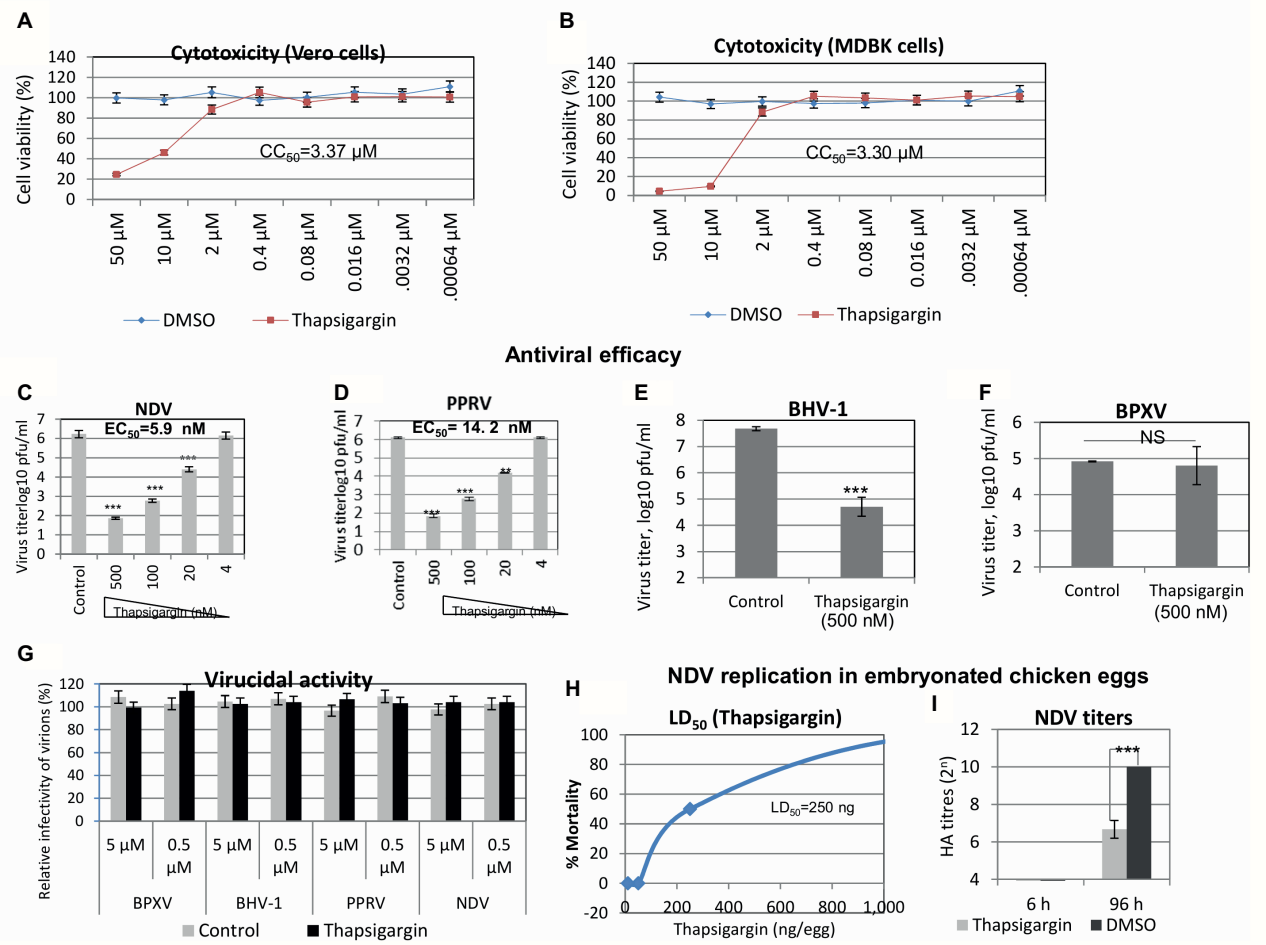
Antiviral efficacy of Thapsigargin. *Cytotoxicity (MTT assay):* five-fold serial dilutions of Thapsigargin (50–0.00064 μM) or equivalent volume of vehicle control (0.05% DMSO) were incubated with cultured Vero/MDBK cells for 96 h, and percentage of cell *via*bility was measured by MTT assay. Cell viability of Vero **(A)** and MDBK **(B)** cells is shown. *Antiviral efficacy*: Vero/MDBK cells were infected with respective viruses at MOI = 0.1 in the presence of indicated concentration of Thapsigargin or vehicle control (0.05% DMSO). Progeny virus particles released in the supernatants were quantified by plaque assay. Antiviral efficacy of Thapsigargin against NDV **(C)**, PPRV **(D)**, BHV-1 **(E)**, and BPXV **(F)** is shown. *Virucidal activity*: 10-fold dilutions of the Thapsigargin or DMSO were mixed with ~10^6^ pfu of the indicated viruses and incubated for 1.5 h at 37°C. Thereafter, the residual viral infectivity was determined by plaque assay in Vero (NDV, PPRV, and BPXV) or MDBK (BHV-1) cells **(G)**. *Effect of Thapsigargin on replication of NDV in embryonated chicken eggs.* Embryonated SPF chicken eggs, in triplicates, were inoculated with indicated concentration of Thapsigargin *via* allantoic route. At 96 h post-Thapsigargin inoculation, eggs were visualized for viability of the embryos. LD_50_ was determined by the Reed-Muench method **(H)**. To analyze the effect of Thapsigargin on NDV replication *in ovo*, embryonated SPF chicken eggs, in triplicates, were infected with NDV *via* allantoic route, along with administration of Thapsigargin (10 ng/egg). At 6 and 96 hpi, the allantoic fluid was examined for NDV yield by HA **(I)**. Error bars indicate SD. Pair-wise statistical comparisons were performed using Student’s t test (** = *p* < 0.01, *** = *p* < 0.001). NS represents no statistical significance.

Thapsigargin is a potent inhibitor of SERCA (Rogers et al., 1995; Eastman and Fidock, 2009). It was identified from a protein kinase inhibitor library described previously (Kumar et al., 2011a). In order to determine the *in vitro* efficacy of Thapsigargin against paramyxoviruses (PPRV and NDV) and DNA viruses (BHV-1 and BPXV), we first determined its cytotoxicity in cultured Vero/MDBK cells by MTT assay. As shown in Figures 1A,B, Thapsigargin at concentrations between 0.00064 and 2 μM did not significantly affect the cell viability even when incubated in cell cultures for 96 h. However, at higher concentrations (>2 μM), it was found to be toxic to the cells. The CC_50_ was determined to be 3.37 and 3.30 μM, respectively, for Vero and MDBK cells. At 2 μM, Thapsigargin cytotoxicity levels varied between 0 and 18% (from experiment to experiment). However, no absolute cytotoxicity was detected at <1 μM (data not shown). Therefore, a highest sub-cytotoxic concentration of 0.5 μM was determined using subsequent experiments.

In order to determine the *in vitro* antiviral efficacy of Thapsigargin, we measured the yield of infectious PPRV and NDV in the presence of indicated concentrations of the Thapsigargin or vehicle control (0.05% DMSO). Thapsigargin significantly inhibited NDV (Figure 1C) and PPRV (Figure 1D) replication (paramyxoviruses) in a dose-dependent manner at an EC_50_ of 5.9 and 14.2 nM, respectively. The therapeutic index (CC_50_/EC_50_) was determined to be 571.18 and 237.32, respectively, for NDV and PPRV. Thapsigargin also inhibited BHV-1 (Figure 1E) but not BPXV (Figure 1F) replication (DNA viruses), suggesting its antiviral efficacy against paramyxoviruses and BHV-1 virus.

Furthermore, in order to determine whether the antiviral efficacy of Thapsigargin against paramyxoviruses is partially due to direct inactivation of the cell free virions, we incubated the infectious virions with either 0.5 or 5 μM Thapsigargin for 1.5 h and subsequently tested the residual infectivity on Vero/MDBK cells. Thapsigargin did not exhibit any virucidal effect on any of the prototype virus tested (Figure 1G), suggesting that the antiviral activity of Thapsigargin is due to the inhibitory effect on virus replication in the target cells.

We also analyzed the effect of Thapsigargin on replication of NDV in embryonated SPF chicken eggs. The lethal dose 50 (LD_50_) of the Thapsigargin was determined to be 250 ng/egg (Figure 1H). At a noncytotoxic concentration of Thapsigargin (10 ng/egg) and at 96 hpi, NDV yield (allantoic fluid) was significantly lower in Thapsigargin-treated eggs, as compared to the vehicle control-treated eggs (Figure 1I), suggesting that Thapsigargin has the potential to inhibit virus replication *in vivo.* There was no detectable virus (HA titer <4) at 6 hpi (Figure 1I) in both Thapsigargin-treated and control-treated eggs, suggesting that the decreased viral titers in Thapsigargin-treated eggs at 96 hpi are actually due to inhibition in virus replication, not simply due to Thapsigargin toxicity.

### SERCA Facilitates Paramyxovirus Replication

**Figure 2:**
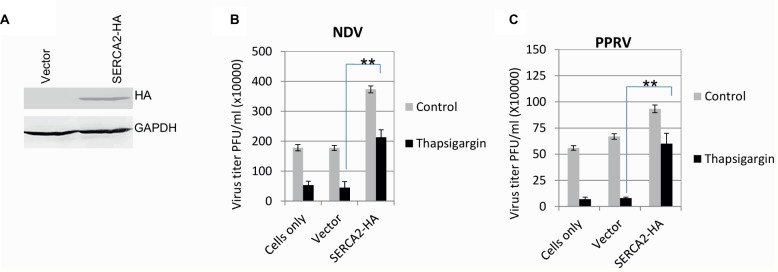
SERCA facilitates paramyxovirus replication. Evaluation of the expression of recombinant SERCA2: HeLa cells were transfected with either an empty vector or with a construct expressing SERCA2-HA. At 48 h-post transfection, cell lysates were probed for expression of HA in Western blot analysis **(A)**. Effect of overexpression of SERCA on paramyxovirus replication: HeLa/goat kidney cells were transfected with SERCA-expressing plasmid (pCR3-SERCA2) or control plasmid (pCR3, empty vector). At 48 h post-transfection, cells were infected with virus (NDV to HeLa cells and PPRV to goat kidney cells) at MOI of 1. Virus released in the supernatant at 24 hpi (NDV) **(B)** or 96 hpi (PPRV) **(C)** was quantified by plaque assay. Error bars indicate SD. Pair-wise statistical comparisons were performed using Student’s t test (** = *p* < 0.01).

In order to further confirm the role of SERCA in virus replication, the inhibitory effect of Thapsigargin was rescued by overexpression of the SERCA. The expression of exogenous/recombinant SERCA was confirmed by probing HA Tag (present at 3′ end of the SERCA) in Western blot analysis (Figure 2A). As compared to the control plasmid (empty vector)-transfected cells, overexpression of SERCA2-HA not only facilitated NDV and PPRV replication but also rescued the inhibitory effect of Thapsigargin on virus replication, suggesting that SERCA2 supports paramyxovirus replication (Figures 2B,C).

### Paramyxoviruses Induce SERCA Expression

**Figure 3:**
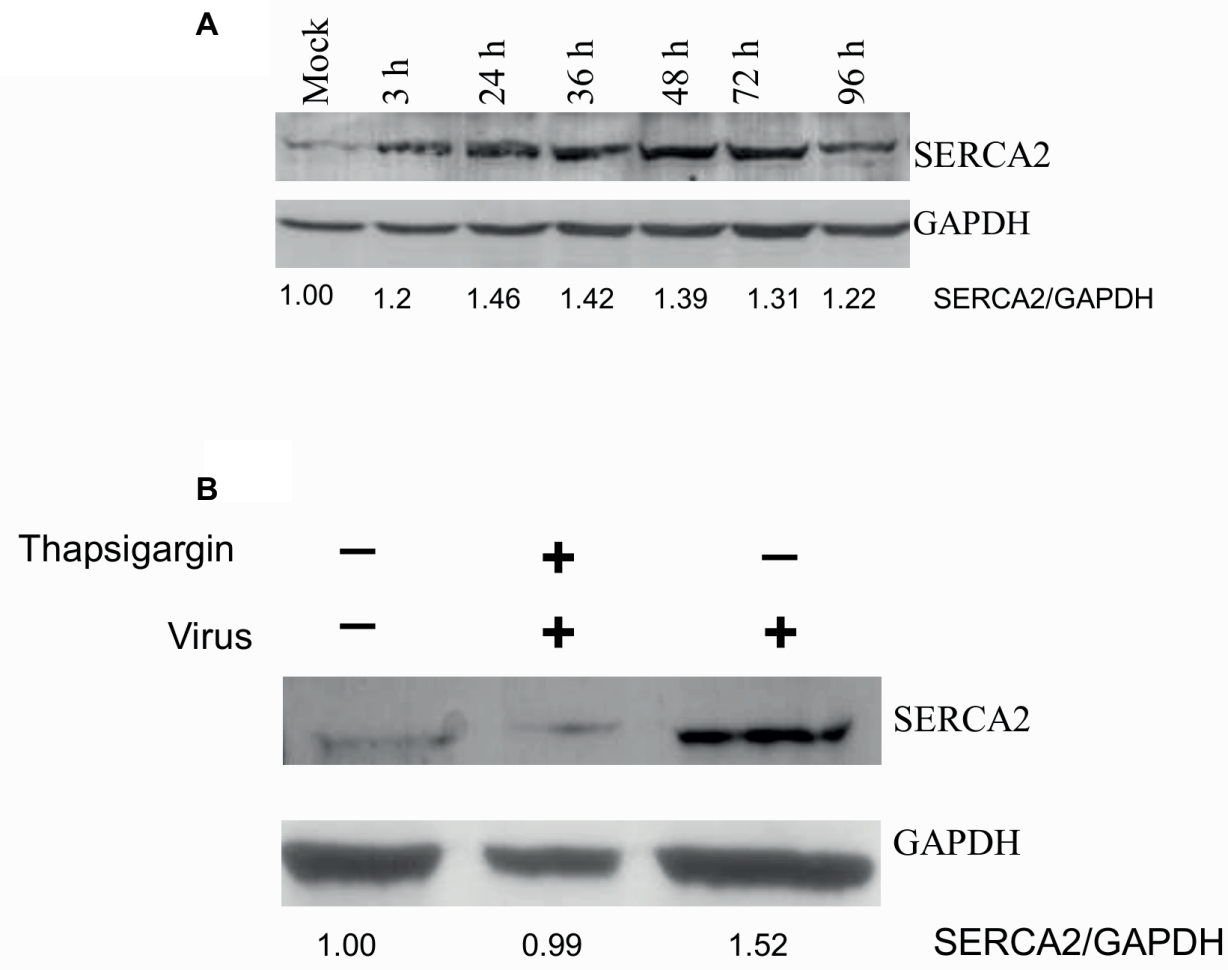
Paramyxovirus infections induce SERCA expression. **(A)** *PPRV infection induces SERCA2 expression:* Vero cells were infected with PPRV at MOI 5, and the cell lysates were prepared at indicated time points. The levels of SERCA2 (upper panel) or house-keeping control protein (GAPDH) (lower panel) were examined by Western blot analysis. **(B)** *Thapsigargin inhibits NDV-induced SERCA2 expression in Vero cells:* Vero cells were infected with NDV at MOI of 5 for 1 h followed by washing with PBS and addition of fresh medium containing Thapsigargin or vehicle control (0.05% DMSO). Cell lysates were prepared at 16 hpi to probe SERCA2 and GAPDH by Western blot analysis. Relative fold-change in the levels of viral/cellular proteins was determined by ImageJ (NIH).

SERCA2 is expressed by most cell types. We also evaluated whether virus infection induces any alteration in SERCA expression. PPRV infection of Vero cells resulted in enhanced SERCA2 expression. As compared to mock-infected cells, a significant induction in SERCA2 expression was observed at 3 hpi, which remained at the peak level between 24 and 72 hpi, before beginning to decline at 96 hpi (Figure 3A, upper panel). However, the levels of house keeping control gene (GAPDH) were similar at all the time points, suggesting that the enhanced levels of SERCA2 expression were related to viral infection (Figure 3A, lower panel). Besides, we also observed that NDV-induced SERCA2 expression could be blocked by Thapsigargin treatment (Figure 3B).

### Time-of-Addition Assay

**Figure 4:**
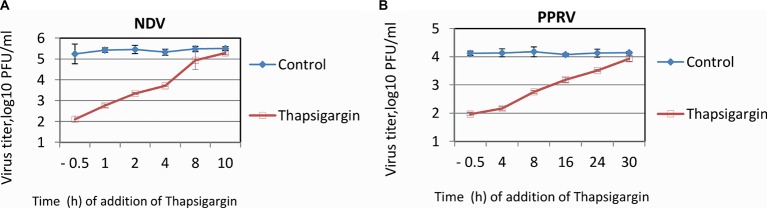
Time-of-addition assay. Confluent monolayers of Vero cells were infected, in triplicate, with PPRV or NDV at MOI of 5, washed six times with PBS and fresh medium with either 0.5 μM Thapsigargin or 0.05% DMSO were added at indicated times. Supernatants were collected at 12 (NDV) or 48 hpi (PPRV) and quantified by plaque assay. Time-of-addition assay for NDV **(A)** and PPRV **(B)** is shown.

In order to ascertain the stage(s) of the viral life cycle which can be impaired by Thapsigargin, we performed a time-of-addition assay (one-step growth curve), in which the inhibitor was applied at different times post-infection, and the virus released into the supernatant was quantified by plaque assay. The NDV and PPRV vary in their length of replication cycle, ~10 and ~24 h, respectively; therefore, time-of-addition of inhibitor and time of virus harvest varied from virus to virus. As shown in Figure 4A, the magnitude of viral (NDV) inhibition gradually decreased. The highest inhibition was observed when the inhibitor was applied 30 min before infection. The inhibition levels progressively decreased from 1 to 6 hpi. Thapsigargin did not exhibit any inhibitory effect on virus replication if it was applied at 10 hpi, a later time point in NDV life cycle when the virus is presumably undergoing budding. Similar findings were observed with PPRV; highest inhibition in viral titers was observed when the inhibitor was applied 30 min prior to infection, magnitude of inhibition progressive decreased from 4 to 24 hpi (Figure 4B). The time-of-addition assay, therefore, suggested that Thapsigargin may inhibit multiple prebudding steps of paramyxovirus replication.

### Effect of Thapsigargin on Specific Steps of Viral Life Cycle

#### Attachment

To analyze the effect of Thapsigargin on attachment of the virus on cell surface, virus was allowed to adsorb (attach) at 4°C (to restrict the entry) in the presence or absence of Thapsigargin. We did not observe any significant difference in the viral titers (adsorbed onto cell surface) between Thapsigargin and vehicle control-treated cells (data not shown), suggesting that Thapsigargin has no effect on attachment of virus to the host cells.

#### Entry

**Figure 5:**
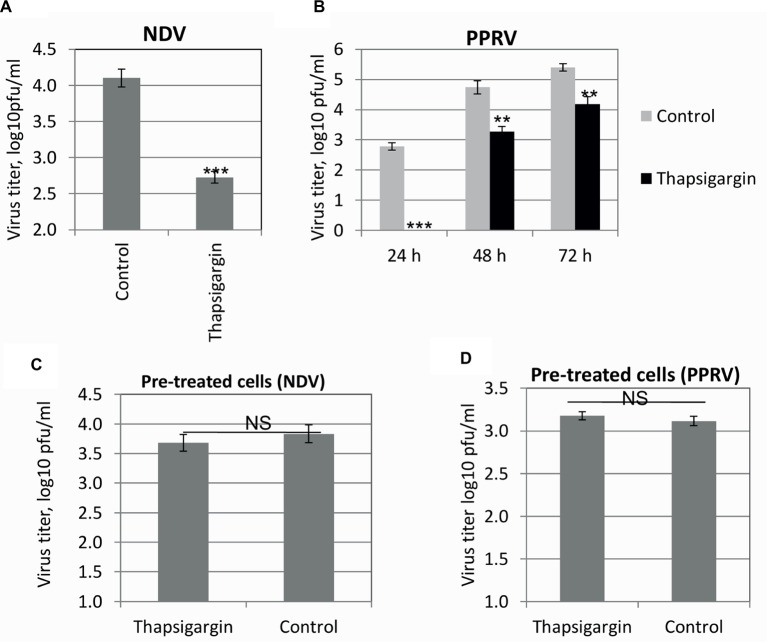
Viral entry. Vero cell monolayers were prechilled to 4°C and infected with the respective viruses at MOI of 5 in Thapsigargin-free medium for 1 h at 4°C to permit the attachment, followed by washing and addition of fresh medium containing Thapsigargin or vehicle control. Entry was allowed to proceed at 37°C for 1 h after which the cells were washed again with PBS to remove any extracellular viruses and incubated with cell culture medium without any inhibitor. The progeny virus particles released in the cell culture supernatants in the treated and untreated cells were titrated by plaque assay. Entry assay for NDV **(A)** and PPRV **(B)** is shown. Vero cells were pretreated with Thapsigargin or DMSO for 1 h followed by washing with PBS and infection with NDV/PPRV at MOI of 5. NDV **(C)** and PPRV **(D)** released in the supernatant were quantified by plaque assay. Error bars indicate SD. Pair-wise statistical comparisons were performed using Student’s t test (** = *p* < 0.01, *** = *p* < 0.001, NS = nonsignificant difference).

In order to determine whether the pre-attached virus was able to enter into the cells in the presence of Thapsigargin, a standard entry assay was performed. Pre-attached virus (4°C) was allowed to enter at 37°C in the presence of Thapsigargin, and infectious virus released in the cell culture supernatant was measured. Thapsigargin treatment resulted in reduced NDV (Figure 5A) and PPRV (Figure 5B) titers, suggesting that SERCA inhibitor blocks paramyxovirus entry.

In order to ascertain that SERCA functions were restored after withdrawal of the Thapsigargin from the cell culture medium (during entry assay) and did not affect post-entry steps of the viral life cycle, we performed an additional experiment where cells were pretreated with Thapsigargin for 1 h at 37°C followed by its removal by washing with PBS. The cells were then infected with NDV/PPRV and grown in the absence of the inhibitor. There was no significant difference in the viral titers between Thapsigargin- and DMSO-pretreated cells in both NDV (Figure 5C) and PPRV (Figure 5D), suggesting that the SERCA functions might have been restored after the removal of Thapsigargin from the cell culture medium.

#### RNA and Protein Synthesis

**Figure 6:**
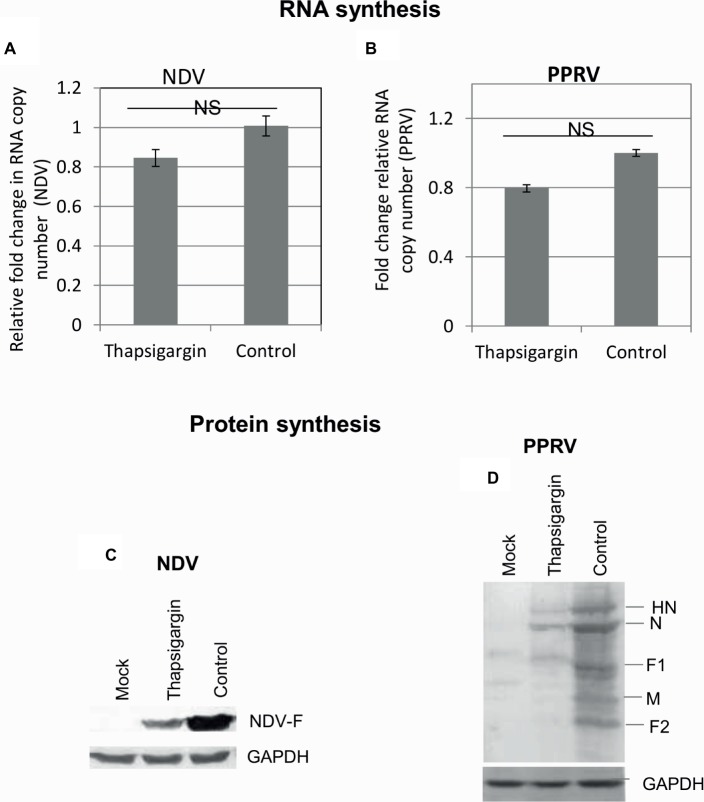
RNA and protein synthesis. Vero cells were either mock-infected or infected with the indicated viruses at MOI of 10 for 3 h followed by washing with PBS and addition of 0.5 μM Thapsigargin or vehicle control (0.05% DMSO). The cells were scrapped at 9 and 20 hpi, respectively, for NDV and PPRV to prepare the cell lysate. Viral RNA was quantified by qRT-PCR. Ct values were normalized with β-actin house-keeping control gene, and relative fold-change was calculated by ΔΔCt method. Relative fold-change in RNA copy number of NDV **(A)** and PPRV **(B)** is shown. The levels of viral proteins were analyzed by Western blot analysis. The levels of viral proteins in NDV **(C)** and PPRV **(D)** infected cells are shown. NS = No significant difference.

In order to determine the effect of Thapsigargin on the synthesis of viral genome/protein, Thapsigargin was applied to the virus-infected cells when early steps of the virus life cycle (attachment/entry) were expected to occur (>3 h). Cell lysates were prepared at 9 and 20 hpi, respectively, for NDV and PPRV to examine the levels of viral RNA and proteins. No significant difference was observed in viral RNA copy number between Thapsigargin- and control-treated cells (Figures 6A,B), suggesting that Thapsigargin has no impact on the synthesis of paramyxoviral genome. However, as compared to the vehicle control (0.05% DMSO)-treated cells, lower levels of viral [NDV (Figure 6C) and PPRV (Figure 6D)] proteins were observed in Thapsigargin-treated cells.

#### Budding

We also analyzed the potential effect of Thapsigargin on the release (budding) of progeny virus particles from infected cells. In budding assay, Thapsigargin was applied at the time when the virus presumably starts budding (during logarithmic phase but before attaining a stationary phase viz; 10 and 36 hpi, respectively, for NDV and PPRV). Viral titers in the supernatants were comparable in Thapsigargin-treated and control-treated cells (data not shown), suggesting that Thapsigargin has no impact on the release of the virus from infected cells.

#### SERCA Inhibition Results in Enhanced Formation of Cytoplasmic Foci in Virus-Infected Cells

**Figure 7:**
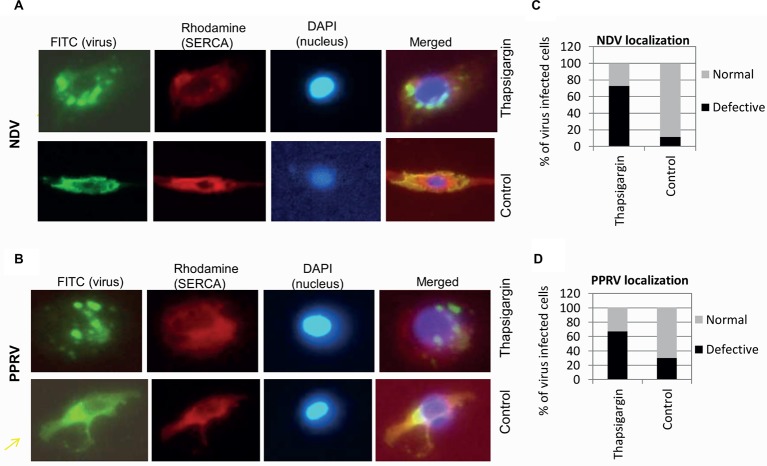
Thapsigargin treatment induces enhanced formation of cytoplasmic foci. Vero cells at ~20% confluence were infected with NDV or PPRV at MOI of 10. At 3 (NDV) or 10 hpi (PPRV), cells were incubated with either 0.5 μM Thapsigargin or 0.05% DMSO. At 10 (NDV) or 36 hpi (PPRV), cells were fixed and subjected for immunofluorescence assay for localization of the viral proteins in the virus-infected cells. Virus was stained with FITC (green), whereas SERCA was stained with rhodamine conjugate. DAPI was used as nuclear stain. Subcellular localization of NDV **(A)** and PPRV **(B)** is shown. Virus-infected cells (FITC) were usually of two types – with or without punctuate structures. To quantitate the subcellular localization, 100 cells (under several different fields) were randomly counted. The percentage of the cells with or without punctuate structures in Thapsigargin-treated and control-treated is shown for NDV **(C)** and PPRV **(D)**.

To further examine whether SERCA inhibitor impacts other intermediate step(s) of viral replication, immunofluorescence assay was performed to monitor the subcellular localization of PPRV and NDV proteins in the cytoplasm of the infected cells. The inhibitor or respective vehicle control (0.05% DMSO) was applied at 3 hpi (NDV) or 10 hpi (PPRV); a time point at which the early events of virus replication (attachment, entry and RNA synthesis) are believed to occur. At 12 hpi (NDV) or 36 hpi (PPRV), when the progeny virus particles presumably bud from the plasma membrane, we observed more number of cytoplasmic foci in Thapsigargin-treated cells, as compared to DMSO-treated cells (Figures 7A,B). Thapsigargin treatment showed cytoplasmic foci in ~70% of the cells, as compared to DMSO control wherein this proportion was 10–30% (Figures 7C,D).

### Selection of Thapsigargin-Resistant Viral Mutants

**Figure 8:**
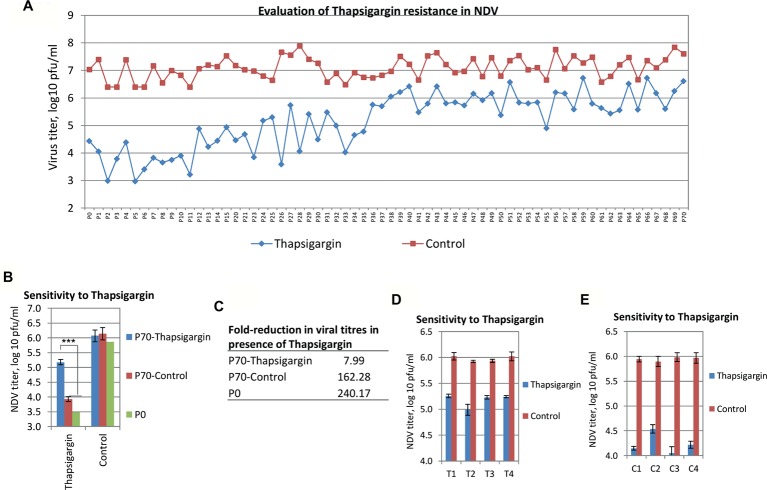
Selection of Thapsigargin-resistant viral mutants. Vero cells were infected with NDV or PPRV at MOI of 0.01 and grown in the presence of either 0.25 μM of Thapsigargin or vehicle control (0.05% DMSO). The progeny virus particles released in the supernatant were harvested either at 72–120 hpi or when ~75% cells exhibited CPE. Seventy (70) such sequential passages were made. **(A)** The levels of NDV inhibition at various passage levels in Thapsigargin-treated and untreated cells are shown. **(B)** S*ensitivity of P70-Thapsigargin NDV to Thapsigargin:* Vero cells, in triplicate, were infected with P0, P70-Thapsigargin, or P70-Control passaged viruses at MOI of 0.1 in the presence of either 0.5 μM Thapsigargin or 0.05% DMSO, and the progeny virus particles released in the supernatant at 24 hpi were quantified. **(C)** Relative growth (fold reduction) of P0, P70-Thapsigargin, and P70-Control viruses in the presence of Thapsigargin. **(D,E)** *Sensitivity of plaque purified (P70) viruses to Thapsigargin:* P70-Thapsigargin or P70-Control viruses were plaque purified [(four plaques each of P70-Control (C1, C2, C3, and C4) and P70-Thapsigargin (T1, T2, T3, and T4)] and amplified in Vero cells. To evaluate the sensitivity to Thapsigargin, plaque purified viruses were infected at MOI of 0.1, and virus yield was measured in the presence of 0.5 μM Thapsigargin or 0.05% DMSO. Error bars indicate SD. Pair-wise statistical comparisons were performed using Student’s t test (*** = *p* < 0.001).

Due to the high genetic barrier to resistance, host-targeting agents provide an interesting perspective for novel antiviral strategies, rather than the directly acting agents. NDV, when passaged sequentially in the presence of a SERCA inhibitor (Thapsigargin, a host-targeting agent), did not generate a completely resistant phenotype against Thapsigargin, even upon 70 passages in Vero cells (Figure 8A). However, resistance began appearing at ~P25 and significant resistance was observed at P35 (~100-fold inhibition compared to ~10,000-fold inhibition at zero passage) after which it became stable without acquiring complete resistance (Figure 8A). As compared to P0 and P70-Control viruses, P70-Thapsigargin virus exhibited significantly lower sensitivity to Thapsigargin, though a completely resistant phenotype could not be observed (Figure 8B). Control-passaged viruses did not exhibit any significant resistance against Thapsigargin even upon 70 passages (Figures 8B,C), suggesting that resistance against Thapsigargin (NDV) is not a general phenomenon due to sequential high passages but rather a specific event acquired in the presence of Thapsigargin.

**Figure 9:**
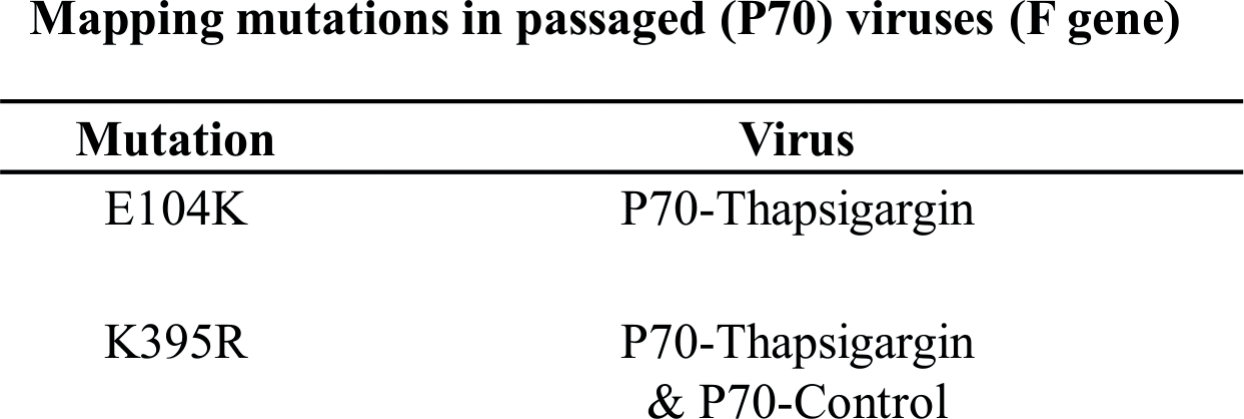
*Nucleotide sequencing of passaged virus.* RNA was extracted from P70-Control and P70-Thapsigargin viruses as well as plaque purified viruses (two plaques each of P70-Control and P70-Thapsigargin) followed by amplification and sequence analysis of F gene. Nucleotide sequences were translated into amino acid sequences and compared with P0 as well as WT sequences retrieved from GenBank (n = 18). Amino acid mutations associated with Thapsigargin resistance as well as those acquired simply due to sequential cell culture passage (present in both P70-Control and P70-Thapsigargin) are shown.

Alternatively, it is possible that the original NDV stock might have contained defective interfering (DI) particles, which suppressed the virus yield. Therefore, we plaque purified P70-Control and P70-Thapsigargin virus stocks – a process which presumably eliminated DI particles. Plaque purified viruses (4 plaques each from P70-Thapsigargin and P70-Control stocks) were again evaluated for their sensitivity to Thapsigargin, wherein P70-Thapsigargin plaques (viruses) were found to replicate at relatively higher titers (~20-fold) (compare Figures 8D,E), as compared to the control viruses, suggesting that the higher growth of P70-Thapsigargin virus was presumably due to mutations in the viral genome, rather than simply due to the suppression of DI particle. Furthermore, we also analyzed the mutations in the F protein of P70-Thapsigargin and P70-Control viruses. As compared to WT [(n = 18, sequences retrieved from GenBank with a global representation)], P0 and P70-Control, P70-Thapsigargin virus showed a unique mutation viz., E104K (Figure 9). In addition, as compared to WT, K395R mutation was present in both P70-Control and P70-Thapsigargin, which might have been simply acquired due to sequential cell culture passages (adaptation) (Figure 9). These mutations were invariably present in both passaged virus stock (P70) and respective plaque purified viruses (Figure 9). However, we could not observe any Thapsigargin-associated mutations in HN gene (data not shown).

## Discussion

High mutation rates in RNA viruses enable resistance to antiviral drugs and preexisting immunity to develop. The rise in incidence of drug resistance has prompted a shift towards the development of novel antiviral drugs. As compared to the viral genome, genetic variability of the host is quite low, and therefore, host-targeting agents are considered to impose a higher genetic barrier to generation of resistant viruses (Fofana et al., 2010, 2012; Lupberger et al., 2011; Khandelwal et al., 2017). Thus, a potentially better approach for the development of novel antiviral therapeutics would be to target host factors required for viral replication, although cytotoxicity of such antiviral agents is of considerable importance. Targeting host factors could have a significant impact on multiple virus genotypes (strain/serotype) and could provide broad spectrum inhibition against different families of viruses which might use the same cellular pathway(s) for replication (Pawlotsky, 2012; Ruiz and Russell, 2012; Conteduca et al., 2014; Shahidi et al., 2014; Khandelwal et al., 2017). This novel approach has led to the development of some promising compounds for the treatment of HCV and HIV (Gilliam et al., 2011; von Hahn et al., 2011). In this study, we have shown that targeting SERCA (a Ca2+ ATPase) by a small molecule chemical inhibitor (Thapsigargin) can block paramyxovirus replication. The inhibitory effect of Thapsigargin could be rescued by overexpression of SERCA, suggesting the virus supportive role of SERCA. Therefore, SERCA may be a novel target for antiviral drug development. Hoffmann and coworkers identified that SPCA1 [a secretary pathway calcium (Ca^2+^) transporter that facilitates Ca^2+^ and Mn^2+^ uptake into the trans-Golgi network] also facilitates replication of the members of the family, *Flaviviridae*, *Togaviridae*, and *Paramyxoviridae* (Hoffmann et al., 2017), suggesting the involvement of multiple calcium transporters in paramyxovirus replication. Mechanistically, SERCA inhibitor was shown to block viral entry and synthesis of viral proteins, besides inducing enhanced formation of cytoplasmic foci. While the precise mechanism of formation of enhanced cytoplasmic foci remains elusive, this might probably have occurred due to general dysregulation of endoplasmic reticulum/altered calcium homeostasis. Furthermore, how SERCA2 regulates both viral entry and synthesis of viral proteins is intriguing and needs further investigation.

It is generally believed that viruses do not acquire resistance against host-targeting antiviral agents (Kumar et al., 2011b, 2014; Pawlotsky, 2012; Chaudhary et al., 2015). However, in a recent study (van der Schaar et al., 2012), Schaar and colleagues identified Coxsackievirus B3 (CVB3) mutants that replicate efficiently in the presence of several potent antiviral drugs known to inhibit phosphatidylinositol-4-kinase IIIα (PI4KIIIα), a key cellular factor for CVB3 replication. The authors observed that a single point mutation in the viral 3A protein confers resistance, and the drug-resistant escape mutants of CVB3 can replicate in cells with low PI4KIIIα. Additionally, cyclosporine A (CsA)-resistant hepatitis C virus (HCV) mutant has also been identified (Coelmont et al., 2009; Chatterji et al., 2010). In our study, resistance acquired by NDV against SERCA inhibitor adds another example to a short list of viruses, which can acquire resistance to host-targeting antiviral agents. To the best of our knowledge, this is the first documented example wherein a paramyxovirus significantly bypasses its dependency on a cellular factor that is targeted by a small molecule inhibitor. While not yet understood, one possible mechanism underlying acquisition of drug resistance is due to change in host factor requirement (Hopcraft and Evans, 2015). For example, under selection pressure in CLDN1 (tight junction protein claudin-1, which serves as an entry factor for HCV) knock-out cells, CLDN1-dependent HCV evolved to use alternate host factors – CLDN6 or CLDN9 (Hopcraft and Evans, 2015). Alternatively, resistant viruses may simply have enhanced affinity for its natural substrate, thereby allowing the virus to propagate despite reduction in concentration of the cellular factors (Kaufmann et al., 2018).

We mapped at least one mutation (E104K) in F protein of Thapsigargin-resistant NDV. Further studies on recombinant NDV harboring point mutation(s) in F and/or other viral proteins are required to precisely understand the mechanism underlying acquisition of drug resistance against Thapsigargin. Though a complete Thapsigargin-resistant NDV phenotype could not be achieved even up to passage level 70, it is a matter of conjecture as to how NDV became less dependent on SERCA (under the selection pressure of Thapsigargin). In immunofluorescence assay, we could not observe a perfect co-localization of SERCA and viral proteins. A co-immunoprecipitation assay to detect a direct interaction between SERCA and virus was also unsuccessful; therefore, in this study, we could not determine any direct interaction between SERCA and viral proteins.

To conclude, we have provided strong evidence for SERCA as a crucial host factor in facilitating optimal paramyxovirus replication, thus validating this as a candidate drug target for the development of antiviral therapeutics. The drug resistance against host-targeting antiviral agents is not an unprecedented event.

## Author Contributions

NKu, SB, SSh, TR and BT designed the experiments. NKu, NKh, RK, YC, KR and KC performed the experiments. NKu, SB, SSh, SSi and BT wrote the manuscript.

### Conflict of Interest Statement

The authors declare that the research was conducted in the absence of any commercial or financial relationships that could be construed as a potential conflict of interest.
